# Glyoxalase System in the Progression of Skin Aging and Skin Malignancies

**DOI:** 10.3390/ijms22010310

**Published:** 2020-12-30

**Authors:** Silvia Yumnam, Lalita Subedi, Sun Yeou Kim

**Affiliations:** 1 College of Pharmacy, Gachon University, 191, Hambakmoero, Yeonsu-gu, Incheon 21936, Korea; silviayumnam@gmail.com (S.Y.); subedilali@gmail.com (L.S.); 2 Gachon Institute of Pharmaceutical Science, Gachon University, Yeonsu-gu, Incheon 21565, Korea

**Keywords:** glyoxalase, methylglyoxal, glyoxal, skin aging, melanoma

## Abstract

Dicarbonyl compounds, including methylglyoxal (MGO) and glyoxal (GO), are mainly formed as byproducts of glucose metabolism. The main glyoxalase system consists of glyoxalase I and II (Glo1 and Glo2) and is the main enzyme involved in the detoxification of dicarbonyl stress, which occurs as an accumulation of MGO or GO due to decreased activity or expression of Glo1. Dicarbonyl stress is a major cause of cellular and tissue dysfunction that causes various health issues, including diabetes, aging, and cancer. The skin is the largest organ in the body. In this review, we discuss the role of the glyoxalase system in the progression of skin aging, and more importantly, skin malignancies. We also discuss the future prospects of the glyoxalase system in other skin abnormalities such as psoriasis and vitiligo, including hyperpigmentation. Finally, in the present review, we suggest the role of glyoxalase in the progression of skin aging and glyoxalase system as a potential target for anticancer drug development for skin cancer.

## 1. Introduction

Dicarbonyl stress is the abnormal accumulation of highly reactive α-oxoaldehydes, such as methylglyoxal (MGO), glyoxal (GO), 3-deoxyglucosone (3-DG), and other dicarbonyl metabolites, leading to cell and tissue dysfunctions causing various health issues, including diabetes, renal failure, aging, and cancer. This is mainly due to the imbalance in the formation and metabolism of dicarbonyl metabolites via glucose metabolism. Another cause of dicarbonyl stress is exposure to exogenous dicarbonyls such as honey. The glyoxalase system in the cytoplasm is the main enzyme that metabolizes MGO and reactive dicarbonyls to D-lactate. The glyoxalase system thus plays an important role in suppressing dicarbonyl stress and maintaining the dicarbonyl metabolites at low tolerable levels, preventing protein and cell dysfunction. As a result, highly reactive α-oxoaldehydes are detoxified enzymatically by the glyoxalase system in the body.

The skin is the largest organ in the body and is the first line of defense against pathogens or environmental changes. The mammalian skin comprises two main layers: dermis and epidermis. The dermis layers consist of fibroblast cells and extracellular matrix, whereas the epidermis layer is the outermost layer of the skin tissue, and through the basement membrane, it connects to the dermis. Keratinocytes are the main epidermal cells and are responsible for renewing tissues. There are some enzymes involved in glucose and glycogen metabolism in the skin epidermis. Among them, glyoxalase is present in epidermal keratinocytes and dermal fibroblasts, and plays a crucial role in skin homeostasis [1]. In the present review, we provide an overview of the role of glyoxalase in the progression of skin aging and skin malignancies. Additionally, we have also discussed glyoxalase as a potential target for anticancer drug development for skin cancer.

### 1.1. Glyoxalase SYSTEM

**Scheme 1:**
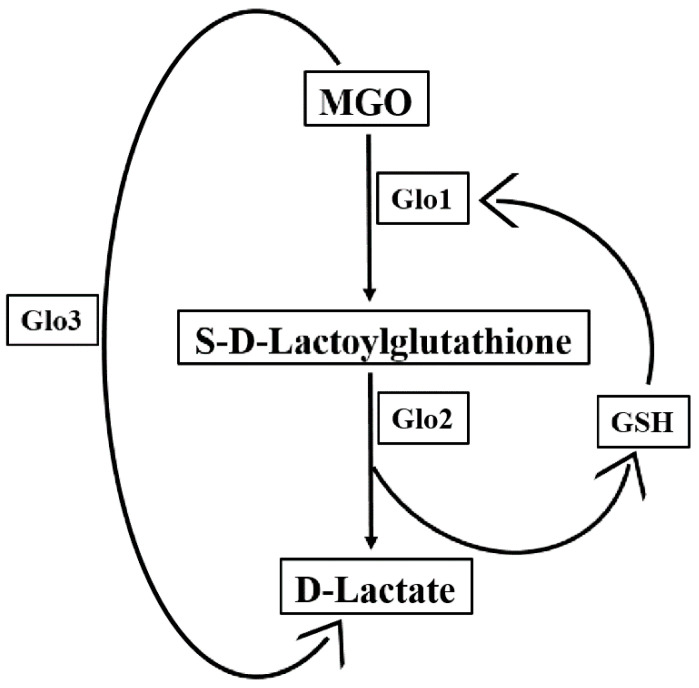
Schematic representation of the glyoxalase system. Glyoxalase I (Glo1) and glyoxalase II (Glo2) are the two main enzymes of the glyoxalase. Scheme 1 uses L-glutathione (GSH) as a co-factor and converts α-oxoaldehydes such as methylglyoxal (MGO) to the corresponding hemithioacetal S-D-lactoylglutathione. Glo2 further catalyzes the hydrolysis of S-D-lactoylglutathione to D-lactate with the regeneration of GSH, which is consumed in the Glo1 catalyzed reaction. Glyoxalase III (Glo3) is a recently discovered glyoxalase enzyme and it directly converts MGO to D-lactate in the absence of any cofactor. Glo1, glyoxalase I; Glo2, glyoxalase II; Glo3, glyoxalase III; MGO, methylglyoxal; GSH, L-glutathione.

The glyoxalase system consists of two intracellular enzymes, glyoxalase I (Glo1) and II (Glo2) (Scheme 1). It was independently discovered by Dakin and Dudley and Neuberg in 1913. Glo1 uses L-glutathione (GSH) as a cofactor and catalyzes the conversion of α-oxoaldehydes such MGO into corresponding hemithioacetal S-d-lactoylglutathione. Glo2 catalyzes the hydrolysis of S-d-lactoylglutathione to D-lactate with the regeneration of GSH, which is consumed in the Glo1 catalyzed reaction. Thus, Glo1 is the rate-limiting step of this series of reactions. Glyoxalase enzymes are present in the cytosol of the cell and are ubiquitously found in all animals. Recently, glyoxalase III (Glo3) was discovered in *Escherichia coli* and it converts MGO to lactic acid in the absence of any cofactor. Interestingly, it has been reported that it belongs to the DJ-1 superfamily proteins, which are involved in Parkinson’s disease and oxidative stress. Glo3 dysfunction may sensitize cells to oxidative stress and induce mitochondrial dysfunction such as DJ-1 [2,3].

### 1.2. Glo1

Glo1 is present in almost all living forms, including animals, plants, yeast, bacteria, and protozoa. The human Glo1 enzyme is a zinc metalloenzyme with two polypeptide chains surrounding an integral zinc atom at their center [4]. It is mainly present in the cytosol of cells. Human GLO-1 is located on chromosome 6 at locus 6p21.2 [5]. In addition to MGO, some of the substrates of Glo1 are GO, hydroxypyruvaldehyde, hydroxypyruvataldehydphosphate, phenylglyoxal, 4,5-dioxovalerate, alkyl-, and arylglyoxales [6,7]. Glo1 is a key enzyme in the anti-glycation defense as it prevents the accumulation of MGO and other reactive dicarbonyls, thereby suppressing the dicarbonyl-mediated glycation reactions. In normal human plasma, the level of dicarbonyl compounds ranges from 50–150 nM and 1–4 μM in mammalian cells. Accumulation of MGO above these levels causes various health issues such as diabetes, renal failure, and aging in both plants and humans. Thus, Glo1 maintains the dicarbonyl metabolites at low tolerable levels, preventing protein and cell dysfunction. The Glo1 promoter region contains various regulatory elements and can regulate both transcriptional and post-translational modifications. Activator protein-2α (AP-2α); early gene 2 factor isoform 4 (E2F4); nuclear transcription factor-κB (NF-κB); activator protein-1 (AP-1), and antioxidant response (ARE), metal-response (MRE), and insulin-response (IRE) elements are some of the transcriptional regulators of Glo1. Among these, AP-2α, E2F4, nuclear factor erythroid 2-related factor 2 (Nrf2), and NF-κB are shown to enhance the Glo1 promoter and upregulate Glo1 expression. The post-translational modifications of Glo1 have been described occur through phosphorylation, nitrosylation, and glutathionylation.

### 1.3. Glo2

Human Glo2 is encoded by the hydroxyacglutathione gene and is located on chromosome 16 in the region 16p13.3. It has two isoforms: the mitochondrial form with a molecular mass of 33.8 kDa and a cytosolic form with a molecular mass of 29.2 kDa; however, both isoforms have the same isoelectric point of 8.3 [8]. It is a monomeric catalyst containing a binuclear Fe(II)Zn(II) metallic center. The catalytic activity is linked to the Zn(II) site, whereas the Fe(II) site has no effect on the catalytic activity [9]. Cytosolic Glo2 has two domains: the active site containing the metal ion binding site and the substrate site. The expression of Glo2 is controlled by the transcription factors p63 and p73 [10], the p53–p21 axis, and androgen receptors [11]. In prostate cancer, phosphatase and tensin homologue/phosphoinositide 3-kinase (PI3K)/protein kinase B (AKT)/mammalian target of rapamycin signaling regulates both Glo1 and Glo2 expression [12].

### 1.4. Glo3

Glo3 was first identified and purified from *Escherichia coli*. It uses α-ketoaldehydes, MGO and phenylglyoxal as substrates and directly converts to D-lactate without GSH [3]. Glo3 expression increases during the stationary phase in *E. coli* and is regulated by RNA polymerase sigma factor [13]. In humans, the DJ-1 homolog, which is involved in Parkinson’s disease and oxidative stress, is responsible for the conversion of MGO to D-lactate without GSH [2]; however, the identification of human Glo3 is still missing.

## 2. Methylglyoxal and Glyoxal

**Figure 1:**
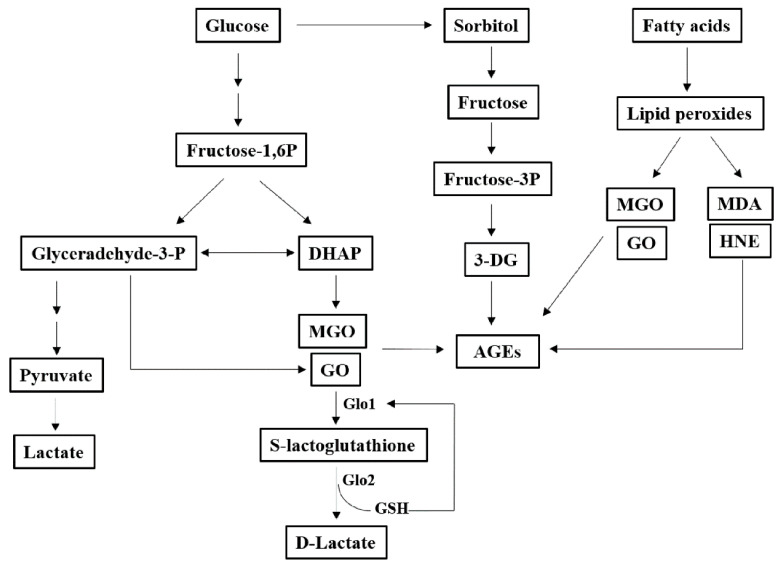
Formation of dicarbonyls and advanced glycation end products (AGEs) from glucose and fatty acid intermediates. During glycolysis, glucose is converted into pyruvate, which subsequently forms lactate. Glycolysis intermediates, glyceraldehyde-3-phosphate, and dihydroxyacetone phosphate (DHAP) are degraded to glyoxal (GO) and MGO, respectively. The glyoxalase system further detoxifies MGO and GO. MGO, GO, and other dicarbonyl compounds including malondialdehyde (MDA) and 4-hydroxynonena (HNE) are formed during lipid peroxidation via degradation of lipid peroxides. Furthermore, 3-deoxyglucosone (3-DG) is formed from fructose-3-phosphate through the polyol pathway. These dicarbonyl compounds form AGEs by reacting with proteins, lipids, and nucleic acids. AGEs, advanced glycation end products; DHAP, dihydroxyacetone phosphate; MGO, methylglyoxal; GO, glyoxal; 3-DG, 3-deoxyglucosone; MDA, malondialdehyde; HNE, 4-hydroxynonena; Glo1, glyoxalase I; Glo2, glyoxalase II.

MGO is mainly formed as a byproduct of glycolysis. The majority of MGO in cells is formed from the degradation of glyceraldehyde-3-phosphate and dihydroxyacetone phosphate [14,15] as shown in Figure 1. It is also formed during lipid peroxidation [16,17] and catabolism of threonine and acetone [18]. GO is mainly formed during lipid peroxidation, degradation of monosaccharides, saccharide derivatives, and glycated proteins. During lipid peroxidation of polyunsaturated fatty acids apart from MGO and GO, lipid peroxides are broken down to other reactive dicarbonyl compounds such as malondialdehyde and 4-hydroxynonena (HNE) [19]. Both MGO and GO are the substrates of Glo1 and are highly potent glycating agents. Apart from glyoxalase, MGO and GO are also metabolized by aldo–keto reductases (AKRs) and aldehyde dehydrogenases (ADHs). However, glyoxalase is the major enzyme in the dicarbonyl stress defense system. Dicarbonyl stress occurs as a result of the accumulation of MGO due to reduced Glo1 activity or expression. During stress, nuclear factor erythroid 2-related factor (Nrf2) controls the basal and inducible expression of Glo1, AKR, and ADH through AREs. Nrf2 enhances the expression and activity of these enzymes, thereby preventing dicarbonyl stress. The binding of MGOs to Kelch-like ECH-associated protein 1 (Keap1) disrupts the nuclear translocation of Nrf2, which is necessary for Glo1 activation. MGO inhibitors suppress MGO toxicity in cells via the Nrf2/Keap1 pathway by increasing GSH levels and promoting MGO metabolism through the glyoxalase system [20].

Glycation of proteins, also known as the “Maillard reaction”, is a complex series of sequential reactions during which reducing sugars such as glucose and fructose react spontaneously with amino residues of proteins, lipids, and nucleic acids leading to the formation of advanced glycation end products (AGEs) including hydroimidazolones, argpyrimidine, tetrahydropyrimidine, Nε-(1-carboxyethyl) lysine (CML), and 1,3-di(Nε-lysino)-4-methyl-imidazolium [21]. During the Maillard reaction, reactive α-dicarbonyls or oxoaldehydes are formed as intermediate products during the Amadori rearrangement. α-Oxoaldehydes include MGO, GO, and 3-DG [22]. In addition to the non-oxidative rearrangement and hydrolysis of Amadori adducts, 3-DG is formed from fructose-3-phosphate in the polyol pathway (Figure 1) [23]. As described above, MGO is formed during anaerobic glycolysis and oxidative decomposition of polyunsaturated fatty acids. α-Oxoaldehydes such as MGO, GO, and 3-DG are all formed during the glycation process by the degradation of glucose or Schiff’s base or as an intermediate in the Amadori rearrangement. These dicarbonyl compounds are highly reactive, causing protein crosslinks and forming AGEs [24]. Some of the other characterized in vivo AGEs are glucosepane, carboxyethyl-lysine (CEL), fructose-lysine glyoxal-lysine dimer (GOLD), and methylglyoxal-lysine dimer (MOLD) [21]. AGEs bind and activate receptors for advanced glycation end products (RAGE), a multiligand receptor of the immunoglobin superfamily [25]. Elevation in MGO and GO causes dicarbonyl stress, which leads to AGE formation and oxidative stress. AGE-RAGE signaling has been linked to inflammation and oxidative stress, which contribute to various health problems, including diabetes, aging, atherosclerosis, and cancer [26,27]. Activation of AGE-RAGE signaling induces the generation of reactive oxygen species and increases the release of inflammatory transcription regulators, including NF-κB, STAT3, and hypoxia-inducible factor 1α (HIF1α). As a result, secretion of inflammatory cytokines such as IL-6, IL-1β, TNF-α, cell adhesion molecules (VCAM1, ICAM1, and endothelin1), and vascular endothelial growth factor is elevated, causing inflammation and angiogenesis. In addition to AGEs, some of the known ligands of RAGE are S100 proteins, high mobility group box 1, prion, and Mac-1 [28]. Previously, many studies have reported that an increase in Glo1 expression and activity attenuates MGO-induced oxidative stress and AGEs production in kidney cells [29,30]. Anti-glycation is considered effective in controlling various diseases such as diabetes, neurological disorders and also slowing senescence and aging. Thus, glyoxalase plays a significant role in anti-glycation.

## 3. Glyoxalase in Skin Aging and Wound Healing

Aging is a progressive change at the cellular, tissue, and organ level, which eventually leads to death. Skin aging is a result of both intrinsic chronological aging and extrinsic photoaging, which is mainly because of UV exposure. Chronologically aged skin appears dry with fine wrinkles and thinning of epidermal and dermal layers. The density of keratinocytes in the basal layer, melanocytes, and Langerhans cells decreases progressively. The extracellular matrix is considerably lost in the aged dermis with increased levels of collagen-degrading metalloproteinases. The photoaged skin appears dry and wrinkled with irregular pigmentation. The epidermal layer becomes thicker, and because of abnormal deposition of elastin material in the dermis, dermal elastosis occurs. Photodamaged skin frequently displays abundant inflammatory cells in the dermis, whereas collagen and elastic fibers show degenerative changes [1]. During skin aging, many modifications in the proteins occur, including oxidative glycation, leading to accumulation of AGEs. The formation of AGEs has been linked to diabetes and other metabolic diseases; however, recently, AGE formation has also been associated with aging. The role of AGE in skin aging has been described previously [31].

As mentioned above, AGEs are formed when proteins react with MGO and GO. In humans and mice, Glo1 expression changes as life progresses. The glyoxalase system is found in the epidermis and dermis of the skin. Radjei et al. [1] demonstrated that glyoxalase enzymes are differentially expressed in the epidermis with Glo1 mainly localized in the basal layer of the epidermis, whereas Glo2 is expressed in the differentiated upper keratinocyte layer in both young and aged skin. Expression of both glyoxalases is more in the aged skin. Interestingly, during chronological aging, the expression of Glo1 was reversed in the basal layer of the epidermis, implying that overexpression of Glo1 may act as a protective mechanism where it protects the skin progenitor cells from the basal epidermal layer against aging-dependent protein modifications, which also explains the lesser accumulation of CML-modified AGE. In addition, Glo2 expression was drastically reduced and the level of CML-modified proteins was increased in photo-exposed old skin; however, Glo1 expression did not change. Lower production of Glo2 with no change in Glo1 expression was also observed in young photo-exposed skin. Even though the expression of Glo1 was not changed, the activity of Glo1 was reduced in keratinocytes from a young donor during senescence and chronological aging. These findings indicate that the glyoxalase system plays an important role in both chronological and photo-exposed aging and acts as a defense system against dicarbonyl stress and oxidative damage [1]. In an in vitro experiment, Glo1 was expressed in both young and old human keratinocytes, and the expression of Glo1 in HaCaT cells was regulated by the Nrf2 gene [1,32].

Aging delays the wound healing process, and patients with diabetes have impaired wound healing. In both aging and diabetic conditions, protein damage due to glycation increases with decreased expression and activity of Glo1. Age-related impairment of wound healing is related to the decreased activity and expression of Glo1. In an in vivo mouse model, old mice showed impaired wound healing with decreased expression and activity of Glo1 compared to young mice. However, there was no significant difference in MGO derived AGEs (MGO-derived hydroimidazolone and CEL). This impaired wound healing capacity in old mice was restored by treatment with aminoguanidine, an MGO scavenger. Treatment with a Glo1 inhibitor also reduced the wound healing capacity in young mice, which indicates that regardless of age, inhibition of Glo1 delays wound healing [33]. These findings indicate that MGO plays a determinant role in wound healing, and the balance between MGO and Glo1 is an important factor in wound healing, regardless of age. Increased accumulation of MGO in diabetic skin collagen has also been reported. Delayed wound healing in diabetic patients is associated with keratinocyte dysfunction and increased accumulation of MGO [34]. Under hyperglycemic conditions, exposure of keratinocytes to MGO suppressed cellular migration. Yang et al. reported that in an in vitro diabetic model, exposure of HaCaT human keratinocyte cells to MGO induced cellular dysfunction and decreased cell adhesion and migration. These conditions were attenuated by treatment with NSHD-1, an H_2_S releasing molecule. Pre-treatment with NSHD-1 improves cell adhesion and wound healing capacity induced by MGO [35]. MGO is accumulated during aging and is also the main cause of delayed wound healing in aged skin. Thus, MGO scavengers and glyoxalase inducers maybe be helpful in delaying the aging process and also hastening the wound healing process.

## 4. Glyoxalase in Skin Malignancies

Skin cancer is one of the most commonly occurring cancers worldwide. They are divided into two types: melanoma and non-melanoma skin cancer. Basal cell carcinoma (BCC) and squamous cell carcinoma (SCC) belong to the non-melanoma group of skin malignancies. Melanoma or cutaneous melanoma is the most aggressive form of skin cancer and accounts for more than 80% of skin cancer-related deaths. Among melanomas, 35–50% have mutation in the BRAF (v-Raf murine sarcoma viral oncogene homolog) gene, 10–25% at the neuroblastoma RAS viral oncogene homolog (NRAS), and 15% had loss of function mutation affecting neurofibromin 1 [36]. Melanomas, similar to other cancer cells, exhibit the Warburg effect and are highly glycolytic. The Warburg effect or aerobic glycolysis is when the cancer cells metabolize glucose into lactate regardless of the oxygen level and supply the necessary ATP for survival and proliferation. Thus, the expression and secretion of lactate dehydrogenase is an important prognostic marker in metastatic melanoma.

In melanomas, activation of the MAPK pathway increases transcription of HIF1α and v-MYC avian myelocytomatosis viral oncogene homolog (MYC). Activation of HIF1α enhances glycolysis as it activates pyruvate dehydrogenase kinase (PDK), which inhibits pyruvate dehydrogenase (PDH), preventing pyruvate from entering the tricarboxylic acid cycle for use in oxidative phosphorylation, OXPHOS. MYC also increases glycolysis by activating lactate dehydrogenase, glucose transporter 1 (GLUT1), and hexokinase 2. Inhibition of microphthalmia-associated transcription factor (MITF) by MAPK pathway activation suppresses OXPHOS and enhances glycolysis [37]. MGO, a dicarbonyl compound that is a byproduct of glycolysis, is highly accumulated in tumor cells, including melanoma. MGO induces the formation of AGEs that are implicated in several pathologies, including diabetes, aging, and cancer. In addition to MGO, reactive carbonyl species such as GO and malondialdehyde are produced in high levels in melanoma resulting in the glycation of proteins, which has been linked to the proliferation and metastases of melanoma. MGO and GO are metabolized by the cellular enzyme glyoxalase. As an adaptive response to elevated MGO stress, cancer cells are known to overexpress glyoxalase enzymes. Glo1 is also overexpressed in both melanoma [38,39,40] and non-melanoma cells [41]. As glyoxalase activity is increased in tumor cells, glyoxalase has become a potential therapeutic target in tumorigenesis.

### 4.1. Glo1 in Melanoma

Glo1 is the major enzyme involved in the detoxification of MGO. Glo1 plays a dual role in cancer. In the non-malignant state, Glo1 acts as tumor suppressor, whereas in the highly metastatic stage with increased glycolytic flux, it promotes tumor growth and proliferation [42]. Overexpression of Glo1 in many cancer cells acts as an adaptive response to high glycolytic activity and elevated MGO, which is often associated with cancer cell survival and resistance to chemotherapeutic agents [42]. Elevated expression of Glo1 is associated with gastric cancer, breast sarcoma, ovarian cancer, prostate carcinoma, and hepatocellular carcinoma [43,44,45,46]. In cancer cells, Glo1 inhibitors are considered potential anticancer targets. Due to the high glycolytic flux of the tumor cells, inhibition of Glo1 causes MGO burst and depletion of GSH, leading to carbonyl and oxidative stress, which eventually leads to cell dysfunction, apoptosis, and necrosis [47]. Glo1 regulates tumor cell survival and proliferation through different mechanisms. AP-2α, E2F4, and Nrf2 are some of the regulators of Glo1 and are highly expressed in tumor cells, and thus induce Glo1 overexpression [48]. Glo1 activates the PI3K/Akt pathway by enhancing NF-κB and AP-1 activities. Activation of the PI3K/Akt pathway increases cell survival and proliferation [49,50]. In addition, the abnormal expression of Glo1 MGO metabolism is accelerated, which in turn inhibits tumor cell apoptosis. Moreover, increased levels of MGO and AGEs activate the p38/MAPK pathway and inhibit NF-κB signaling. This, in turn, increases the expression of pro-apoptotic proteins, Bax and p53, and decreases that of anti-apoptotic proteins including XIAP, survivin, Bcl-2, and Bcl-xL [48,51]. Thus, amplification of Glo1 activity detoxifies intracellular MGO and GO, which consequently promotes tumor cell survival, proliferation, angiogenesis, and invasion. Increased Glo1 expression is also associated with multidrug resistance in cancer chemotherapy. Glo1 overexpression protects tumor cells against cytotoxicity induced by anti-tumor drugs, whereas inhibition of Glo1 expression overcomes multidrug resistance and restores tumor sensitivity to anti-tumor drugs [42,52].

Recently, Bair et al. reported a 4- to 10-fold increase in Glo1 expression in metastatic melanoma compared to that in healthy control cells. His team showed that Glo1 inhibition increased MGO-induced cytotoxicity in human melanoma cells and observed heat shock protein 27 as the target protein of post-translation by MGO in metastatic melanoma cells [38]. Downregulation of Glo1 by miR-137 inhibits melanoma cell proliferation, whereas re-expression of Glo1 reduces the inhibitory effect of miR-127 on melanoma cell proliferation, suggesting that Glo1 expression is essential for melanoma proliferation [40]. Similarly, Jandova et al. also reported that silencing of Glo1 inhibits A375 melanoma cell migration and invasion by modulating epithelial mesenchymal transition-related genes, and this expression was reversed by the re-expression of Glo1 in Glo1 knockout A375 cells. In addition, the tumor migration in SCID (Severe combined immunodeficient) mice induced by A375-Glo1 knockout cells was relatively lower than that in wild-type A375 cells [39]. These studies strongly suggest that Glo1 plays a major role in melanoma tumor invasion and migration.

Among the Glo1 inhibitors, intravenous administration of glycyl, the glutamyl diethyl ether form of S-(N-p-chlorophenyl-N-hydroxycarbamoyl) glutathione (CHG), inhibits melanoma tumor growth. CHG is a potent inhibitor of Glo1, and its rapid release in melanoma tissue inhibits Glo1, causing an increase in intracellular MGO levels, resulting in tumor growth inhibition [53]. Zheng et al. demonstrated that a sulfoxide copolymer of *N*-(2-hydroxypropyl)methacrylamide and/or S-(N-4-chlorophenyl-N-hydroxycarbomyl-thioethyl)methacrylamide is a potent Glo1 inhibitor that inhibits B16 melanoma cell growth. In addition, the antitumor effects of these polymer prodrugs were more potent than those of their unpolymerized counterparts [32]. These studies indicate that Glo1 is highly expressed in melanoma and is involved in melanoma progression and proliferation and inhibition of Glo1 inhibits melanoma progression and invasion, making Glo1 a novel target for melanoma.

### 4.2. Glo1 in Non-Melanoma

Glo1 is overexpressed in SCC, BCC, and verrucous carcinoma tissues, whereas its expression is lower in benign skin neoplasms and normal skin [41]. Silencing of Glo1 in SCC-13 cells inhibits cell proliferation by inhibiting anti-apoptotic proteins such as Bcl-2, Bcl-xL, survivin, and Xiap as well as cell migration and invasion. Moreover, Glo1 siRNA transfection also inhibited the in vivo SCC progression in a xenograft model [41]. In addition to melanoma, non-melanoma SCC and BCC also showed overexpression of Glo1, which makes Glo1 a potential target in skin malignancies. As Glo1 is highly expressed in both melanoma and non-melanoma skin cancer, we proposed that Glo1 is a novel target in skin cancer and more extensive studies are necessary in future animal and clinical studies for future drug development against skin cancer.

### 4.3. Glo2 in Skin Cancer

In contrast to Glo1, the role of Glo2 in skin cancer has not yet been researched. Significant research is needed to investigate the role of Glo2 in skin cancer.

## 5. Glyoxalase in Miscellaneous Skin Abnormalities

Hyperpigmentation is a condition in which the skin becomes darker than the surrounding area. UV exposure intensifies skin pigmentation and in photoaged skin, other than wrinkling, skin hyperpigmentation also occurs [54]. However, to date, the involvement of the glyoxalase system in skin pigmentation and melanogenesis has not been studied. Recently, Lee et al. reported that similar to other skin cells, melanocytes also expressed RAGE, the AGE receptor in both in vitro cells and in vivo tissues. His team demonstrated that incubation of melanocytes with AGE increases melanin production, tyrosinase activity and also increases melanogenesis-associated molecules such as MITF and tyrosinase. In addition, exposure of cultured human skin to AGE increases melanin pigmentation. The increase in melanin production was inhibited by blocking RAGE showing that AGE-induced melanogenesis is through RAGE [55]. Thymoid^®^, a chemically standardized black cumin seed extract, has a higher inhibitory effect of AGE formation than aminoguanidine, an MGO scavenger. Additionally, Thymoid^®^ inhibits melanin production of murine melanocytes, B16F10 cells and also inhibits protein and mRNA expression of melanogenesis related molecular markers—MITF, tyrosinase, tyrosinase-related protein 1 (TRP1) and TRP2 [56]. These studies strongly suggest that AGEs play a role in melanogenesis and their inhibition may have anti-melanogenic effect. However, the direct involvement of glyoxalase in skin pigmentation is not yet studied. It is hoped that in the future, the role of glyoxalase in skin pigmentation and melanogenesis is investigated and explored.

Psoriasis and vitiligo are immune-mediated skin disorders that are genetically inherited. Metabolomic profiling of serum samples from patients with psoriasis showed enhanced amino acid metabolic activity and increased glycolysis with decreased fatty acid biosynthesis compared to that in healthy individuals. High lactic acid levels in patients with psoriasis verify the increased glycolytic activity in psoriasis [57,58]. A comparative study of 40 individuals each with psoriasis and vitiligo with 80 healthy individuals showed no significant difference in the genetic markers of red cell enzymes, including Glo1 and plasma proteins [59]. However, RNA expression profiling of uninvolved and lesion skin from psoriasis patients showed decreased expression of Glo1 [60]. Hong et al. reported that the skin of patients with atopic dermatitis (AD) has a higher expression of AGEs in corneocytes than in normal healthy controls, and the accumulation of dermal AGEs in AD increases oxidative stress, causing skin inflammation [61]. AD is a chronic inflammation of the skin characterized by pruritus and eczema. Gromwell roots suppress skin inflammation and inhibit glycation by increasing Glo1 and glutathione synthesis [62]. The role of glyoxalase in dermatology fields other than skin aging has not been extensively studied. Extensive investigation is needed to study the role of glyoxalase enzymes in various skin abnormalities, including hyperpigmentation, psoriasis, vitiligo, and AD.

## 6. Concluding Remarks

**Figure 2:**
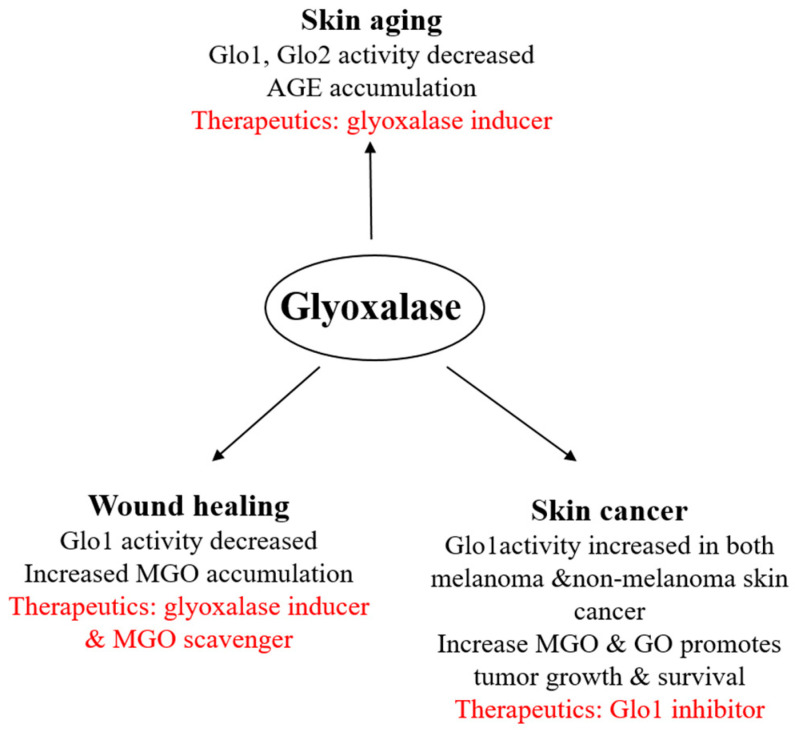
Schematic representation of the glyoxalase system in skin aging, skin cancer and other skin abnormalities. Glyoxalase activity (Glo1 and Glo2) is reduced in skin aging and age-related wound healing with the accumulation of MGO, GO and AGE, while Glo1 activity is highly expressed in both melanoma and non-melanoma skin cancer. With the increase in Glo1, intracellular MGO and GO are detoxified, promoting tumor growth and survival. Glyoxalase regulators are potential therapeutic agents to reduce skin abnormalities. The role of glyoxalase in other skin abnormalities such as hyperpigmentation, psoriasis, vitiligo, AD etc., and Glo2 in skin cancer, has not yet been investigated. Glo1, glyoxalase I; Glo2, glyoxalase II, MGO, methylglyoxal; GO, glyoxal, AD, atopic dermatitis.

The glyoxalase system plays an important role in suppressing dicarbonyl stress and maintaining the dicarbonyl metabolites at low tolerable levels, preventing protein and cellular dysfunction. Therefore, both Glo1 and Glo2 may be a powerful preventive system in the body, particularly through detoxifying harmful dicarbonyl stress produced as a result of glucose metabolism and sun exposure in the skin. Figure 2 represents the role of glyoxalase in skin aging and skin cancer. During skin aging, both chronological and photoaging, the expression and activity of glyoxalase is greatly reduced, which leads to the accumulation of MGO and causes oxidative damage to the skin. This accumulation of MGO also impairs and delays the wound healing process. The glyoxalase system plays an important role in skin aging and protects the skin against dicarbonyl stress and oxidative damage. Thus, glyoxalase inducers or MGO scavengers may play an important role in delaying skin aging and improving the wound healing process. Although the role of glyoxalase has been studied broadly in skin aging, its role in skin pigmentation and other skin abnormalities such as psoriasis, vitiligo, and AD still needs to be studied and explored. Future studies on the role of glyoxalase in skin pigmentation and other skin abnormalities might reveal the role of this protein in various skin disorders. Hence, it is highly recommended in the future to explore the role of glyoxalase in various skin disorders. Due to the high expression of Glo1 in skin malignancies, Glo1 has become a novel target for the development of drugs against skin cancer. Further research on glyoxalase both in in vivo and clinical studies in skin carcinogenesis might reveal interesting roles of these proteins in skin cancer, and more efforts are also needed to develop potential glyoxalase regulators to reduce skin abnormalities, including skin aging and skin melanoma.

## Data Availability

Data is contained within the article.
